# Phenogrouping and risk stratification of patients undergoing cardiac resynchronization therapy upgrade using topological data analysis

**DOI:** 10.1038/s41598-023-47092-x

**Published:** 2023-11-23

**Authors:** Walter Richard Schwertner, Márton Tokodi, Boglárka Veres, Anett Behon, Eperke Dóra Merkel, Richárd Masszi, Luca Kuthi, Ádám Szijártó, Attila Kovács, István Osztheimer, Endre Zima, László Gellér, Máté Vámos, László Sághy, Béla Merkely, Annamária Kosztin, Dávid Becker

**Affiliations:** 1 Heart and Vascular Center, Semmelweis University, 68 Városmajor Street, 1122 Budapest, Hungary; 2 Cardiac Electrophysiology Division, Department of Internal Medicine, University of Szeged, Szeged, Hungary

**Keywords:** Cardiology, Cardiac device therapy

## Abstract

Choosing the optimal device during cardiac resynchronization therapy (CRT) upgrade can be challenging. Therefore, we sought to provide a solution for identifying patients in whom upgrading to a CRT-defibrillator (CRT-D) is associated with better long-term survival than upgrading to a CRT-pacemaker (CRT-P). To this end, we first applied topological data analysis to create a patient similarity network using 16 clinical features of 326 patients without prior ventricular arrhythmias who underwent CRT upgrade. Then, in the generated circular network, we delineated three phenogroups exhibiting significant differences in clinical characteristics and risk of all-cause mortality. Importantly, only in the high-risk phenogroup was upgrading to a CRT-D associated with better survival than upgrading to a CRT-P (hazard ratio: 0.454 (0.228–0.907), *p* = 0.025). Finally, we assigned each patient to one of the three phenogroups based on their location in the network and used this labeled data to train multi-class classifiers to enable the risk stratification of new patients. During internal validation, an ensemble of 5 multi-layer perceptrons exhibited the best performance with a balanced accuracy of 0.898 (0.854–0.942) and a micro-averaged area under the receiver operating characteristic curve of 0.983 (0.980–0.986). To allow further validation, we made the proposed model publicly available (https://github.com/tokmarton/crt-upgrade-risk-stratification).

## Introduction

Chronic, high-burden right ventricular pacing (RVP) has detrimental effects on cardiac structure and function and is associated with an increased risk of adverse outcomes regardless of the pre-implantation left ventricular (LV) systolic function^1–6^. If also accompanied by a decline in LV systolic function compared to baseline and no alternative trigger can be identified, this condition is termed pacing-induced cardiomyopathy^7^. Although the underlying pathophysiologic mechanisms have not been fully elucidated yet, it has been presumed that inter- and intra-ventricular dyssynchrony are the primary culprits^7,8^. RVP fundamentally perturbs electrical activation (i.e., the activation pattern becomes similar to the one seen in patients with left bundle branch block)^9^, leading to impaired mechanical contraction^10,11^, which then results in abnormal myocardial metabolism, altered regional perfusion, increased fibrosis, functional mitral regurgitation, reduced cardiac output, and increased filling pressures^7,8,12–14^. By ameliorating the extent of dyssynchrony, upgrading to cardiac resynchronization therapy (CRT) may reverse the deleterious consequences of RVP, even after very long periods of RVP^15–18^. Importantly, patients with no history of ventricular arrhythmias (VAs) and an LV ejection fraction (LVEF) of ≤ 35% fall also under the indications for implanting an implantable cardioverter-defibrillator (ICD) for primary prevention^19^. Nevertheless, it is still challenging to determine which patients would exhibit an additional benefit from upgrading to a CRT-defibrillator (CRT-D) in lieu of a CRT-pacemaker (CRT-P).

The current pacing guidelines of the European Society of Cardiology (ESC) provide recommendations and guidance to aid physicians in choosing between a CRT-D and a CRT-P^20^. Nevertheless, these were primarily intended for facilitating the selection of the optimal device in patients undergoing de novo CRT implantation, and as shown by the European CRT Survey II and several other studies, patients referred for CRT upgrade differ from those referred for de novo CRT implantation^21–23^. Thus, further evidence is required to determine whether clinical characteristics and risk factors should be considered with the same weight in the risk assessment and device selection of candidates for CRT upgrade as for de novo CRT implantation. Given the complexity of risk stratification and optimizing device selection, novel data analysis techniques, such as topological data analysis (TDA) and machine learning (ML), could play a pivotal role in these tasks as they are aptly suited for the integrated and personalized assessment of risk profiles^24–27^.

Accordingly, we sought to apply TDA to identify phenogroups of patients with previously implanted pacemakers (PMs) and no history of VAs, in whom upgrading to a CRT-D is associated with better long-term survival than upgrading to a CRT-P. We also trained ML classifiers to enable the classifications of new patients into the identified phenogroups, hence facilitating the selection of CRT upgrade candidates who would benefit from choosing a CRT-D over a CRT-P device.

## Methods

### Study cohort, data collection, and ethical approval

We retrospectively identified patients with conventional PMs who were referred for a CRT-P or CRT-D upgrade at the Heart and Vascular Center of Semmelweis University (Budapest, Hungary) between December 2001 and August 2020. Patients with a previously implanted ICD device or a history of VAs were excluded. CRT upgrade procedures were performed as per guidelines. The new LV lead was implanted via subclavian venous access, preferably into the lateral, posterolateral, or posterior tributary of the coronary sinus, as described previously by our research group^28^. In case the implantation of the LV lead was not feasible into any of the coronary sinus branches, transeptal lead implantation was performed^29^. For each patient, pre-upgrade clinical characteristics (i.e., demographics, medical history, cardiovascular risk factors, physical status, currently applied pharmacological therapy, electrocardiographic, echocardiographic, and laboratory results) were retrieved from the electronic medical records system of Semmelweis University.

The study was performed in accordance with the principles outlined in the Declaration of Helsinki. The Regional and Institutional Committee of Science and Research Ethics of Semmelweis University approved the study protocol (approval No. 161-0/2019) and waived the requirement for informed consent due to the retrospective nature of the study.

### Outcome of interest

Outcome data [status (dead or alive), date of death] were obtained for all patients by querying Hungary’s National Health Insurance Database in May 2021. The primary endpoint of our study was death from any cause, and the time to death was measured from the date of the upgrade procedure. Right censoring was applied if a patient (1) was still alive 10 years after the upgrade procedure, (2) had a subsequent CRT-D upgrade after being upgraded to a CRT-P device, or (3) underwent heart transplantation.

### Topological data analysis

TDA can be perceived as an unsupervised ML framework that creates compact and interpretable visual representations of high-dimensional datasets. The key idea behind TDA is that tools of shape analysis can be used to identify and connect data points (e.g., patients) with similar characteristics in a multi-dimensional space and then plot the data as a two-dimensional topological network. The generated network consists of nodes (representing collections of similar patients) connected by edges (i.e., lines between two nodes) if they have at least one patient in common. Networks can be color-coded based on the outcome of interest to gain insight into the data. Two input parameters are required to construct a topological network: (1) a distance metric, which measures the similarity between data points, and (2) one or more lenses, which are filter functions describing the data distribution. Before generating a topological network, the gain (which controls the number of nodes) and resolution (which controls the number of edges) must be defined for each lens.

In this study, we used 16 features to generate the topological network: age, sex, type of the implanted device (CRT-P or CRT-D), New York Heart Association (NYHA) functional class, history of atrial fibrillation (AF), history of hypertension, history of diabetes mellitus (DM), etiology of heart failure (HF), history of myocardial infarction, history of percutaneous coronary intervention, history of coronary artery bypass graft surgery, serum creatinine, glomerular filtration rate (GFR), LVEF, and LV end-diastolic and end-systolic diameters. Missing values of the features were replaced using mean imputation, and then features were Z-score transformed. We applied normalized correlation as the distance metric with two multi-dimensional scaling lenses (with a resolution of 25 and a gain of 2.1, both equalized). Patients placed into nodes not connected to the main network (n = 36) were considered outliers and were omitted from the further steps of the analysis.

After the topological network was created, we wanted to divide it into regions with different clinical characteristics and risks of all-cause mortality. To this end, we first performed community autogrouping using the Louvain method to find the best possible grouping of nodes with high intra-group but low inter-group connectivity^30^. With this algorithm, we generated 14 autogroups, which were then sorted based on the survival rate of their members to identify groups with the lowest and the highest mortality rates. Next, each group was merged with an adjacent group exhibiting the most similar mortality rate. This step was repeated multiple times until three phenogroups (i.e., low-, intermediate-, and high-risk phenogroups) with a nearly equal number of patients were created (Supplementary Fig. 1). Due to the inherent nature of TDA, the phenogroups overlapped partially (i.e., 5 patients belonged to two phenogroups). However, this phenomenon does not violate any assumptions or requirements of the statistical tests used for subgroup comparisons.

TDA and autogrouping were performed using the EurekaAI Workbench (version 3.1.0, SymphonyAI, Palo Alto, California, USA) and the EurekaAI Python SDK (version 3.1.0, SymphonyAI, Palo Alto, California, USA).

### Machine learning models for classifying new patients into the TDA-derived phenogroups

To provide a solution for classifying new patients into the TDA-derived phenogroups, we assigned each patient to one of the three groups based on their location in the topological network and used this labeled data to train several different multi-class classifiers. For training and evaluating such classifiers, we customized our previously published ML framework that was originally designed for binary classification^31^. Training and internal validation were performed using nested cross-validation (with a 5-fold inner cross-validation loop for hyperparameter tuning and a 5-fold outer cross-validation loop for model selection and evaluation), resulting in an ensemble of 5 classifiers that can be applied to the data of new patients. Balanced accuracy was used as the scoring metric, and we also calculated accuracy, micro- and macro-averaged precision, recall, F1 scores, and areas under the receiver operating characteristic curve (AUCs).

The ensemble model exhibiting the best performance during internal validation was also tested in an additional cohort of 29 patients who underwent a CRT upgrade procedure at the Cardiac Electrophysiology Division of the Department of Internal Medicine of University of Szeged (Szeged, Hungary) between September 2005 and August 2020. Outcome data were obtained by querying Hungary’s National Health Insurance Database in August 2023. The study protocol was approved by the Human Investigation Review Board of the University of Szeged (approval No. 4681), with a waiver of informed consent due to the retrospective nature of the study.

ML analysis was performed in Python (version 3.9.13, Python Software Foundation, Wilmington, Delaware, USA). The source code and the best-performing ensemble model are publicly available on GitHub (https://github.com/tokmarton/crt-upgrade-risk-stratification).

### Statistical analysis

Continuous variables are expressed as mean ± standard deviation or median (interquartile range). The characteristics of the CRT-D and CRT-P upgrade groups were compared using unpaired Student’s t-test or Mann–Whitney U test (for continuous variables) and Chi-squared or Fisher’s exact test (for categorical variables), as appropriate. The characteristics of the three TDA-derived phenogroups were compared in a pairwise manner using the Kolmogorov–Smirnov test (for continuous variables) and Chi-squared or Fisher’s exact test (for categorical variables), as appropriate. The survival of subgroups and phenogroups was visualized using Kaplan–Meier curves, and log-rank tests were performed for comparison. Follow-up duration was estimated using the reverse Kaplan–Meier method, and mortality was calculated based on Kaplan–Meier estimates. Univariable and multivariable Cox proportional hazards models were used to compute hazard ratios (HRs) with 95% confidence intervals (CIs). A 2-sided *p*-value of < 0.05 was considered statistically significant. All statistical analysis was performed in R (version 4.1.2, R Foundation for Statistical Computing, Vienna, Austria).

## Results

### Baseline clinical characteristics of the study cohort

Between December 2001 and August 2020, 611 patients underwent CRT upgrade procedures at the Heart and Vascular Center of Semmelweis University. After excluding those with a previously implanted ICD device (n = 224) or a history of VAs (n = 116), the final study cohort included 326 patients, from whom 117 (36%) were upgraded to a CRT-D and 209 (64%) to a CRT-P. The median time between the initial PM implantation and the upgrade procedure was 5.5 (2.2–8.9) years. Before the CRT upgrade procedure, 34 (10%) patients had a VDD, 132 (41%) a VVI, and 160 (49%) a DDD PM. The median RVP rate was 97% (77–100%). During the period with chronic RVP, LVEF decreased by 20 (10–24) percentage points.

The baseline clinical characteristics of the CRT-D and CRT-P upgrade patients are presented in Table 1. Patients upgraded to a CRT-D device were more likely to be males (*p* = 0.011) and had higher GFR (*p* = 0.036), whereas loop diuretics were administered less frequently (*p* = 0.004) in this group than in the CRT-P upgrade group.

**Table 1:** Clinical characteristics of the study cohort.

	All n = 326	Upgraded to CRT-P n = 209	Upgraded to CRT-D n = 117	*p* value
Age, years	73.8 (68.7–78.9)	74.0 (68.8–79.2)	73.6 (68.4–78.1)	0.528
Male	245 (75)	147 (70)	98 (84)	0.011
NYHA functional class III-IV	157 (48)	104 (50)	53 (45)	0.511
Medical history				
Atrial fibrillation	176 (54)	117 (56)	59 (50)	0.396
Diabetes mellitus	122 (37)	78 (37)	44 (38)	1.000
Hypertension	250 (77)	157 (75)	93 (80)	0.448
Ischemic etiology of HF	163 (50)	104 (50)	59 (50)	1.000
Myocardial infarction	116 (36)	78 (37)	38 (33)	0.450
PCI	107 (33)	64 (31)	43 (37)	0.314
CABG	54 (17)	32 (15)	22 (19)	0.510
Time to upgrade, years	5.5 (2.2–8.9)	5.5 (2.0–9.2)	5.4 (2.9–8.9)	0.601
Laboratory parameters				
NT-proBNP, pg/mL (110)	2752 (1534–4666)	2986 (1944–5163)	2616 (1500–4586)	0.496
Creatinine, μmol/L (251)	107 (87–142)	114 (88–146)	101 (86–133)	0.103
GFR, mL/min/1.73m^2^ (251)	58 (44–76)	55 (42–75)	65 (46–77)	0.036
Echocardiographic parameters				
LVIDd, mm (280)	61 ± 8	61 ± 9	60 ± 7	0.636
LVIDs, mm (224)	49 (44–56)	50 (44–57)	49 (45–54)	0.825
LVEF, % (292)	30 (25–35)	30 (25–35)	29 (25–33)	0.108
Medications				
ACE-I/ARB	297 (91)	190 (91)	107 (92)	1.000
Beta-blocker	295 (91)	186 (89)	109 (93)	0.301
Loop diuretics	256 (79)	175 (84)	81 (69)	0.004
MRA	221 (68)	137 (66)	84 (72)	0.301
Amiodarone	56 (17)	33 (16)	23 (20)	0.462

The value in parenthesis after a feature’s name indicates the number of patients with available data. If no value is reported, the given feature is available for all patients. Continuous variables are expressed as mean ± standard deviation or median (interquartile range), whereas categorical variables are reported as frequencies (n) and percentages (%). The characteristics of the CRT-D and CRT-P upgrade groups were compared using unpaired Student’s t-test or Mann–Whitney U test for continuous variables and Chi-squared or Fisher’s exact test for categorical variables, as appropriate.

*ACE-I* angiotensin-converting enzyme inhibitor, *ARB* angiotensin receptor blocker, *CABG* coronary artery bypass graft surgery, *CRT-D* cardiac resynchronization therapy-defibrillator, *CRT-P* cardiac resynchronization therapy-pacemaker, *GFR* glomerular filtration rate, *HF* heart failure, *LVEF* left ventricular ejection fraction, *LVIDd* left ventricular internal diameter at end-diastole, *LVIDs* left ventricular internal diameter at end-systole, *MRA* mineralocorticoid receptor antagonist, *NT-proBNP* N-terminal pro-brain natriuretic peptide, *NYHA* New York Heart Association, *PCI* percutaneous coronary intervention.

### Survival of CRT-D vs. CRT-P upgrade patients

**Figure 1:**
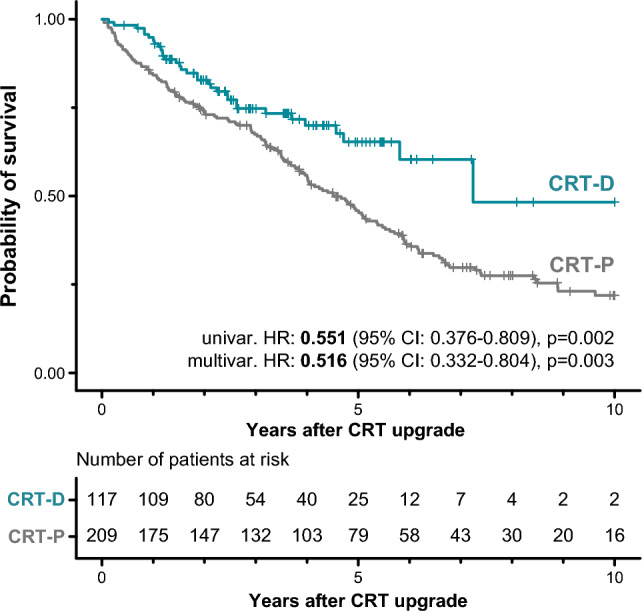
Kaplan–Meier curves depicting the survival of patients upgraded to a CRT-D vs. those upgraded to a CRT-P. Univariable and multivariable Cox proportional hazards models were used to compute hazard ratios with 95% confidence intervals. Besides the type of the implanted device (CRT-D or CRT-P), the multivariable model included the following covariates: age (at the time of the upgrade procedure), sex, history of atrial fibrillation, etiology of heart failure, serum creatinine, left ventricular ejection fraction, angiotensin-converting enzyme inhibitors or angiotensin receptor blockers, and loop diuretics. *CI* confidence interval, *CRT* cardiac resynchronization therapy, *CRT-D* cardiac resynchronization therapy-defibrillator, *CRT-P* cardiac resynchronization therapy-pacemaker, *HR* hazard ratio.

Over the median follow-up of 6.0 (3.7–8.9) years, 178 (55%) patients died in our cohort. Seven (2%) patients with CRT-P had a subsequent CRT-D upgrade, and 2 (1%) patients underwent heart transplantation during the follow-up period. Based on Kaplan–Meier estimates, 5- and 10-year mortality were 49 (43–55)% and 74 (66–80)% in the entire cohort, 35 (23–45)% and 52 (21–71)% in patients upgraded to a CRT-D, and 54 (47–61)% and 78 (70–84)% in those who underwent a CRT-P upgrade, respectively. Upgrading to a CRT-D was associated with a lower risk of all-cause death than upgrading to a CRT-P based on univariable (unadjusted HR: 0.551; 95% CI 0.376–0.809; *p* = 0.002) and multivariable Cox regression analysis (adjusted HR: 0.516; 95% CI 0.332–0.804; *p* = 0.003) as well (Fig. 1, Table 2). Besides the device type, male sex (HR: 2.045; 95% CI 1.209–3.460; *p* = 0.008) and loop diuretics (HR: 1.785; 95% CI 1.061–3.001; *p* = 0.029) were also found to be independent predictors of all-cause death in multivariable Cox regression analysis (Table 2).

**Table 2:** Predictors of all-cause mortality.

	Univariable Cox regression HR (95% CI)	Multivariable Cox regression HR (95% CI)
Age, years	1.031 (1.011–1.051), *p* = 0.003	1.011 (0.987–1.037), *p* = 0.369
Male	1.549 (1.080–2.222), *p* = 0.018	2.045 (1.209–3.460), *p* = 0.008
CRT-D	0.551 (0.376–0.809), *p* = 0.002	0.516 (0.332–0.804), *p* = 0.003
NYHA functional class III-IV	1.294 (0.961–1.743), *p* = 0.090	
Medical history		
Atrial fibrillation	1.364 (1.009–1.844), *p* = 0.044	1.178 (0.806–1.721), *p* = 0.398
Diabetes mellitus	1.265 (0.935–1.710), *p* = 0.127	
Hypertension	0.931 (0.658–1.317), *p* = 0.686	
Ischemic etiology of HF	1.927 (1.420–2.617), *p* < 0.001	1.205 (0.815–1.781), *p* = 0.350
Myocardial infarction	1.941 (1.439–2.619), *p* < 0.001	
PCI	1.485 (1.093–2.017), *p* = 0.011	
CABG	1.253 (0.857–1.832), *p* = 0.244	
Time to upgrade, years	0.979 (0.949–1.009), *p* = 0.168	
Laboratory parameters		
Creatinine (251)	1.004 (1.001–1.007), *p* = 0.004	1.003 (0.815–1.781), *p* = 0.129
GFR (251)	0.990 (0.982–0.998), *p* = 0.011	
Echocardiographic parameters		
LVIDd (280)	1.020 (0.999–1.042), *p* = 0.061	
LVIDs (224)	1.016 (0.996–1.035), *p* = 0.111	
LVEF (292)	0.978 (0.958–0.998), *p* = 0.035	0.979 (0.956–1.003), *p* = 0.084
Medications		
ACE-I/ARB	0.578 (0.365–0.915), *p* = 0.019	0.765 (0.443–1.322), *p* = 0.337
Beta-blocker	0.653 (0.417–1.023), *p* = 0.063	
Loop diuretics	2.004 (1.292–3.108), *p* = 0.002	1.785 (1.061–3.001), *p* = 0.029
MRA	1.066 (0.778–1.461), *p* = 0.692	
Amiodarone	0.971 (0.639–1.475), *p* = 0.890	

The value in parenthesis after a feature’s name indicates the number of patients with available data. If no value is reported, the given feature is available for all patients.

*ACE-I* angiotensin-converting enzyme inhibitor, *ARB* angiotensin receptor blocker, *CABG* coronary artery bypass graft surgery, *CI* confidence interval, *CRT-D* cardiac resynchronization therapy-defibrillator, *GFR* glomerular filtration rate, *HF* heart failure, *HR* hazard ratio, *LVEF* left ventricular ejection fraction, *LVIDd* left ventricular internal diameter at end-diastole, *LVIDs* left ventricular internal diameter at end-systole, *MRA* mineralocorticoid receptor antagonist, *NT-proBNP* N-terminal pro-brain natriuretic peptide, *NYHA* New York Heart Association, *PCI* percutaneous coronary intervention.

**Figure 2:**
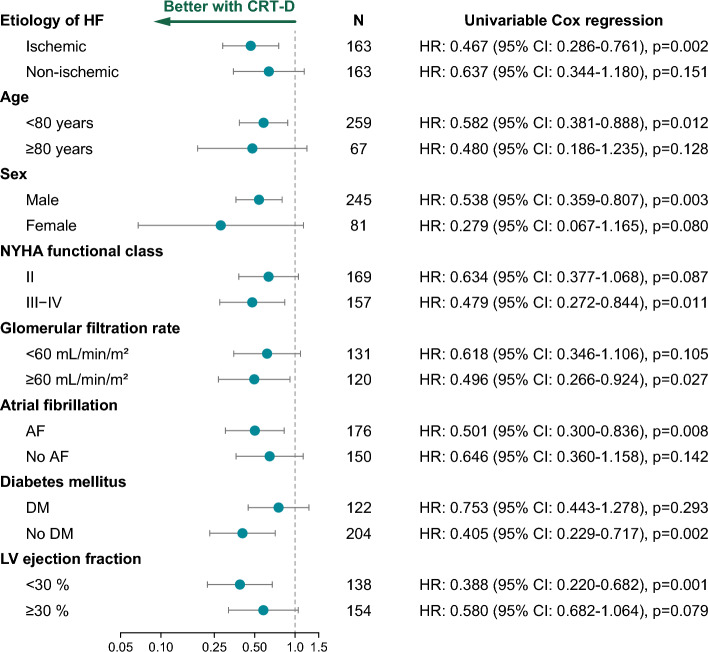
Forest plot summarizing the results of the subgroup analysis. After dividing the study cohort into subgroups based on different clinical characteristics, univariable Cox regression analysis was performed to identify those subgroups in whom upgrading to a CRT-D is associated with better survival than upgrading to a CRT-P. *AF* atrial fibrillation, *CI* confidence interval, *CRT-D* cardiac resynchronization therapy-defibrillator, *CRT-P* cardiac resynchronization therapy-pacemaker, *DM* diabetes mellitus, *HR* hazard ratio, *LV* left ventricular, *NYHA* New York Heart Association.

We also wanted to investigate whether upgrading to a CRT-D is associated with better survival than upgrading to a CRT-P in different subsets of patients. To this end, patients were split into subgroups based on HF etiology (ischemic and non-ischemic), age (< 80 and ≥ 80 years), sex, NYHA functional class (II and III-IV), GFR (< 60 and ≥ 60 mL/min/m^2^), history of AF, history of DM, and LVEF (< 30 and ≥ 30%). Upgrading to a CRT-D was associated with better survival than upgrading to a CRT-P in men, patients with ischemic HF, < 80 years of age, NYHA functional class III-IV, higher GFR, AF, no DM, and < 30% of LVEF (Fig. 2).

Over the past decades, we have witnessed significant advancements in the pharmacological and device therapy of HF, prompting guideline updates on multiple occasions. Nonetheless, we found no association between the year of the CRT upgrade procedure and all-cause mortality using Cox regression.

### Clinical characteristics and outcomes of the TDA-derived phenogroups

**Figure 3:**
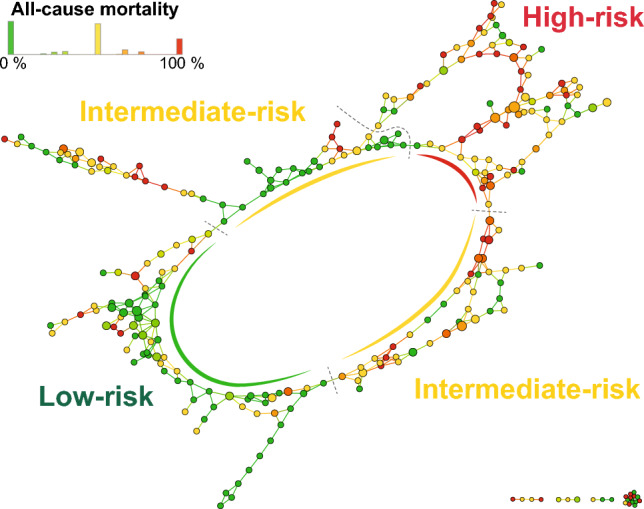
The topological network of patients undergoing CRT upgrade. The topological network was created using 16 pre-upgrade features (metric: normalized correlation, lenses: 2 × multi-dimensional scalings [resolution: 25, gain: 2.10, equalized]). The generated network consists of nodes with edges between them. Each node represents a collection of similar patients, and two nodes are connected if they have at least one patient in common. In this network, nodes are color-coded based on all-cause mortality. The topological network was divided into low-risk, intermediate-risk, and high-risk regions (i.e., phenogroups) based on all-cause mortality. *CRT* cardiac resynchronization therapy.

The application of TDA and autogrouping resulted in a looped network in which the low-risk and high-risk regions were located at opposite poles (Fig. 3). These two regions were conjoined by sections containing patients with an intermediate risk of death on both the lower and upper arc of the loop. The combination of the two intermediate-risk regions is referred to as the intermediate-risk phenogroup throughout the manuscript.

The phenogroups showed several differences in clinical characteristics (Table 3). The proportions of males and patients with ischemic etiology were the highest in the high-risk and lowest in the low-risk phenogroups. Patients in the high-risk phenogroup had the largest LV diameters and the lowest LVEF values, whereas individuals in the low-risk phenogroup had the best renal function.

**Table 3:** Clinical characteristics of the phenogroups.

	Low-risk phenogroup n = 92	Intermediate-risk phenogroup n = 109	High-risk phenogroup n = 94
Age, years*	75.2 (69.4–78.9)	73.8 (66.2–79.1)	72.4 (68.9–78.1)^†^
Male*	53 (58)	82 (75)^†^	87 (93)^†‡^
CRT-D*	42 (46)	31 (28)^†^	33 (35)
NYHA functional class III-IV*	37 (40)	53 (49)	50 (53)
Medical history			
Atrial fibrillation*	59 (64)	54 (50)	45 (48)^†^
Diabetes mellitus*	25 (27)	43 (39)	45 (48)^†^
Hypertension*	72 (78)	82 (75)	70 (75)
Ischemic etiology of HF*	2 (2)	51 (47)^†^	94 (100)^†‡^
Myocardial infarction*	1 (1)	39 (36)^†^	63 (67)^†‡^
PCI*	0 (0)	36 (33)^†^	61 (65)^†‡^
CABG*	1 (1)	21 (19)^†^	28 (30)^†^
Time to upgrade, years	5.7 (2.5–9.3)	6.1 (2.4–11.2)	3.9 (1.7–7.7)^‡^
Laboratory parameters			
NT-proBNP, pg/mL (98)	2,834 (1,548–4,797)	2,847 (1,206–5,211)	3,000 (1,754–5,043)
Creatinine, μmol/L (225)*	96 (80–111)	119 (89–149)^†^	120 (95–151)^†^
GFR, mL/min/1.73m^2^ (225)*	65 (50–80)	53 (38–74)^†^	53 (42–72)^†^
Echocardiographic parameters			
LVIDd, mm (249)*	59 ± 5	60 ± 11^†^	64 ± 7^†‡^
LVIDs, mm (202)*	48 ± 5	49 ± 12^†^	54 ± 7^†‡^
LVEF, % (261)*	30 (28–35)	30 (25–35)	28 (25–32)^†‡^
Medications			
ACE-I/ARB	86 (94)	97 (89)	88 (94)
Beta-blocker	85 (92)	97 (89)	87 (93)
Loop diuretics	67 (73)	86 (79)	82 (87)^†^
MRA	63 (69)	78 (72)	61 (65)
Amiodarone	15 (16)	20 (18)	15 (16)

*Variables used as input features in topological data analysis.

^†^*p* < 0.05 vs. low-risk phenogroup, ^‡^*p* < 0.05 vs. intermediate-risk phenogroup.

The value in parenthesis after a feature’s name indicates the number of patients with available data. If no value is reported, the given feature is available for all patients. Continuous variables are expressed as mean ± standard deviation or median (interquartile range), whereas categorical variables are reported as frequencies (n) and percentages (%). The pairwise comparison of phenogroups was performed using the Kolmogorov–Smirnov test for continuous variables and Chi-squared or Fisher’s exact test for categorical variables, as appropriate.

*ACE-I* angiotensin-converting enzyme inhibitor, *ARB* angiotensin receptor blocker, *CABG* coronary artery bypass graft surgery, *CRT-D* cardiac resynchronization therapy-defibrillator, *GFR* glomerular filtration rate, *HF* heart failure, *LVEF* left ventricular ejection fraction, *LVIDd* left ventricular internal diameter at end-diastole, *LVIDs* left ventricular internal diameter at end-systole, *MRA* mineralocorticoid receptor antagonist, *NT-proBNP* N-terminal pro-brain natriuretic peptide, *NYHA* New York Heart Association, *PCI* percutaneous coronary intervention.

**Figure 4:**
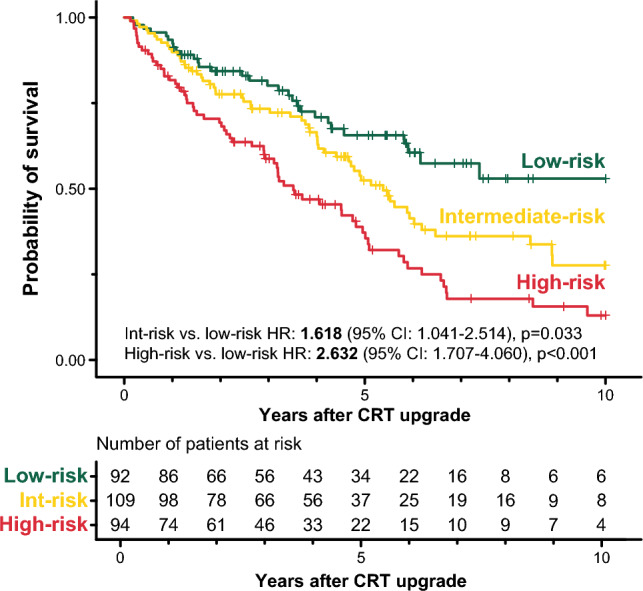
Kaplan–Meier curves depicting the survival of the topological data analysis-derived phenogroups. Hazard ratios and 95% confidence intervals were calculated with univariable Cox regression.. *CI* confidence interval, *CRT* cardiac resynchronization therapy, *HR* hazard ratio.

**Figure 5:**
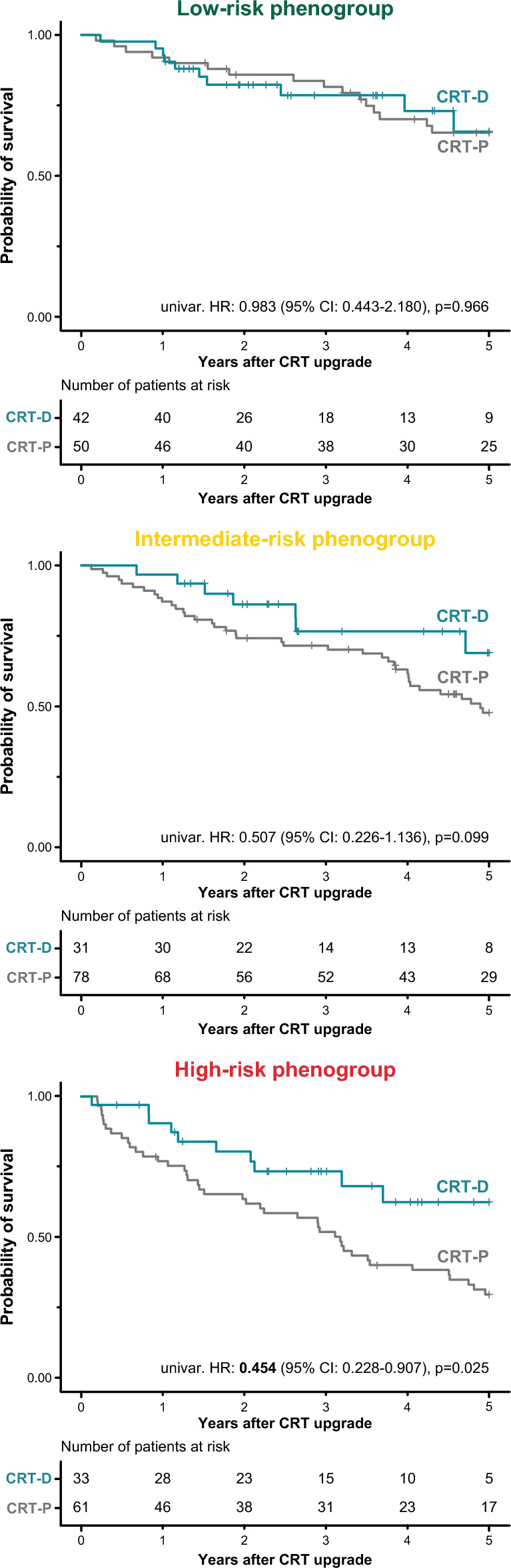
Kaplan–Meier curves depicting the survival of patients upgraded to a CRT-D vs. those upgraded to a CRT-P in each topological data analysis-derived phenogroup. In these plots, right censoring is applied 5 years after the upgrade procedure. Univariable Cox proportional hazards models were used to compute hazard ratios with 95% confidence intervals. *CI* confidence interval, *CRT* cardiac resynchronization therapy, *CRT-D* cardiac resynchronization therapy with defibrillator, *CRT-P* cardiac resynchronization therapy pacemaker, *HR* hazard ratio.

As expected, there were also significant differences in the survival of the phenogroups (log-rank test: *p* < 0.001, Fig. 4). Patients of the intermediate-risk and high-risk phenogroup had a 1.6-fold (unadjusted HR: 1.618; 95% CI 1.041–2.514; *p* = 0.033) and 2.6-fold (unadjusted HR: 2.632; 95% CI 1.707–4.060; *p* < 0.001) increase in the risk of all-cause mortality than those belonging to the low-risk phenogroup, respectively. Compared to upgrading to a CRT-P, upgrading to a CRT-D was associated with a lower risk of death in high-risk patients (unadjusted HR: 0.454; 95% CI 0.228–0.907; *p* = 0.025) but neither in the intermediate-risk (unadjusted HR: 0.507; 95% CI 0.226–1.136; *p* = 0.099) nor the low-risk phenogroups (unadjusted HR: 0.983; 95% CI 0.443–2.180; *p* = 0.966) (Fig. 5).

Since the intermediate-risk phenogroup comprised two separate subgroups—one at the lower arc and the other at the upper arc of the circular network, we also compared their clinical characteristics and survival (Supplementary Table 1). Patients in the upper region were older (*p* < 0.001) and less symptomatic (*p* <0.001). They had predominantly ischemic etiology (*p* < 0.001), lower N-terminal pro-brain natriuretic peptide (NT-proBNP) values (*p* < 0.001), smaller LV end-diastolic and end-systolic diameters (both *p* < 0.001), and higher LVEF values (*p* < 0.001) than those mapped to the lower region. Despite these differences in their clinical characteristics, they had similar survival rates (Supplementary Fig. 2), and upgrading to a CRT-D was associated with a similar risk of all-cause mortality as upgrading to a CRT-P in both the upper (HR: 0.445; 95% CI 0.131–1.510; *p* = 0.194) and the lower intermediate-risk region (HR: 0.546; 95% CI 0.185–1.609; *p* = 0.273) (Supplementary Fig. 3).

### Performance of the multi-class classifiers

**Figure 6:**
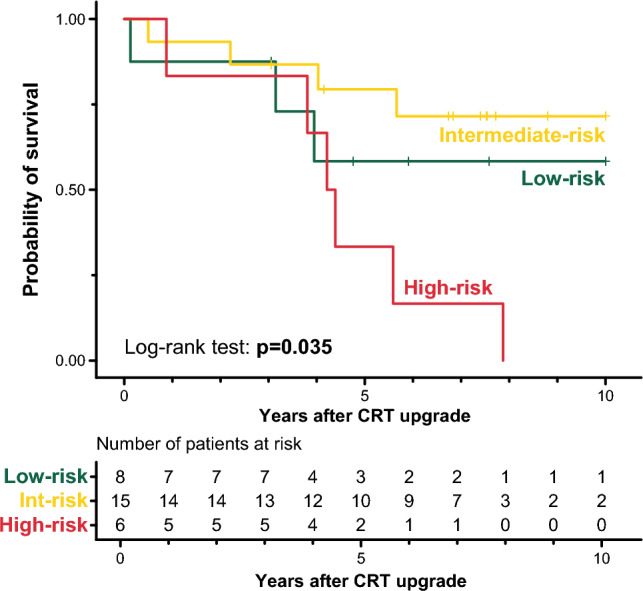
Kaplan–Meier curves depicting the survival of the patients of the external validation cohort classified into the topological data analysis-derived phenogroups. *CRT* cardiac resynchronization therapy.

Among the evaluated multi-class classifiers, the ensemble of 5 multi-layer perceptrons exhibited the best performance during internal validation with a balanced accuracy of 0.898 (95% CI: 0.854–0.942) and a micro-averaged AUC of 0.983 (95% CI: 0.980–0.986) (Supplementary Table 2). In the external validation cohort (see clinical characteristics in Supplementary Table 3), all patients who were classified into the high-risk phenogroup (n = 6) died within 10 years following the upgrade procedure (Fig. 6). Nevertheless, differences between the survival of the three phenogroups were less pronounced, which is most likely attributable to the small sample size.

## Discussion

To the best of our knowledge, this retrospective observational study is the largest to date that investigated patients with previously implanted PMs and no history of VAs who underwent CRT upgrade. Using conventional statistical analysis, we found that CRT-D upgrade was associated with a lower risk of all-cause mortality than CRT-P upgrade, even after adjusting for the relevant clinical covariates. In addition, we applied TDA for the simultaneous evaluation of 16 clinical features and generated a circular topological network in which we could delineate three phenogroups exhibiting significant differences in the risk of all-cause mortality. Interestingly, only in the high-risk phenogroup was upgrading to a CRT-D associated with better survival than upgrading to a CRT-P, implying that choosing a CRT-D over a CRT-P may not convey an additional survival benefit in all CRT upgrade candidates. We also trained and evaluated an ML classifier that can be used to classify new patients into the TDA-derived phenogroups and pinpoint those who might benefit from implanting a CRT-D instead of a CRT-P. To allow other researchers to use the proposed model for research purposes and validate its performance independently, we made it publicly available along with the scripts used for training and validation (https://github.com/tokmarton/crt-upgrade-risk-stratification).

It is well-known that chronic RVP has deleterious effects on cardiac structure and function, presumably due to inducing inter- and intra-ventricular dyssynchrony, and is associated with an increased risk of adverse outcomes^1–6^. By addressing dyssynchrony, upgrading to a CRT may mitigate or even reverse the harmful consequences of chronic RVP, resulting in improved clinical outcomes^32^. However, it is still a matter of debate whether an ICD would exert any additional benefit in patients undergoing a CRT upgrade.

Although CRT upgrade procedures make up 20–30% of all CRT implantations^21^, only a limited number of randomized controlled trials (RCTs) have been conducted in the context of CRT upgrade so far^32,33^. The most recently published one is the BUDAPEST-CRT Upgrade trial, which demonstrated that CRT-D upgrade was associated with a lower incidence of the primary (composite of all-cause mortality, HF hospitalization, or < 15% decrease in LV end-systolic volume at 12 months) and the secondary endpoints (composite of all-cause mortality or HF hospitalization) compared to ICD-only therapy^34^. Nevertheless, no RCTs have been conducted yet to compare CRT-D vs. CRT-P upgrades; thus, we have to rely solely on data from observational studies. In a study investigating non-ischemic patients with no history of VAs upgraded to CRT due to pacing-induced cardiomyopathy, Barra et al. reported a low risk of life-threatening VAs and observed that these patients may not derive any significant benefit in terms of all-cause mortality from the addition of an ICD^35^. In contrast, Leyva et al. observed a lower risk of all-cause mortality after upgrading to a CRT-D than a CRT-P in a cohort including both ischemic and non-ischemic HF patients with no history of VAs, even after inverse probability weighting^36^. The results of these studies emphasize the importance of etiology in device selection and are in line with our observations, as we also found that upgrading to a CRT-D is associated with better survival than upgrading to a CRT-P in the ischemic but not the non-ischemic subgroup of patients.

Numerous observational studies have investigated the impact of CRT-D vs. CRT-P in patients undergoing de novo CRT implantation as well^37,38^. However, the evidence provided by these studies is ambiguous, and no RCT has been published to date that was specifically designed for the head-to-head comparison between CRT-P and CRT-D in the context of de novo CRT implantation. Importantly, the ongoing Re-evaluation of Optimal Re-synchronization Therapy in Patients with Chronic Heart Failure (RESET-CRT) trial, hypothesizing that CRT-P is non-inferior to CRT-D concerning all-cause mortality, is expected to provide crucial data on this matter^39^. As a prelude to this RCT, a population-based weighted cohort study was also conducted with the same inclusion and exclusion criteria and primary endpoint, and the investigators found CRT-P to be non-inferior in terms of survival after adjusting for age and entropy balancing for baseline clinical characteristics^40^. Nevertheless, as these studies have included de novo CRT patients only, further investigations are required to confirm or refute that their results also apply to patients undergoing CRT upgrade, given the apparent differences in clinical characteristics between patients referred for CRT upgrade and those referred for de novo CRT implantation.

As current guidelines lack specific recommendations for guiding device selection during CRT upgrades in patients with previously implanted PMs and no history of VAs^20,41^, physicians must carefully weigh the advantages and drawbacks of upgrading to a CRT-D instead of a CRT-P on an individual basis. During this comprehensive and individualized pre-upgrade assessment (i.e., benefit-risk analysis), multiple factors, such as HF etiology, age, comorbidities, and device-related risks and potential complications, must be evaluated simultaneously^23^. It should also be kept in mind that although patients presenting with an LVEF of 35% or lower fall under the indications for an ICD, CRT may significantly improve LV function, and LVEF may surpass 35%, mitigating the risk of sudden cardiac death (SCD) and obviating the need for an ICD. Furthermore, choosing the optimal device type may be further complicated by the concurrent use of HF medications (e.g., angiotensin receptor-neprilysin inhibitors and sodium-glucose cotransporter 2 inhibitors), which can also independently reduce the incidence of SCD^42^.

Given the challenges and complexity of the pre-upgrade assessment, we sought to apply advanced data analysis approaches in the present study to identify those CRT upgrade candidates who are most likely to experience an additional mortality benefit from an ICD. We decided to use TDA as it can simultaneously evaluate multiple clinical features and construct a compact visual representation of a complex dataset (i.e., a topological network)^43^. Then, through the exploratory analysis of the generated network, distinct phenogroups with different characteristics, clinical outcomes, and therapeutic responses can be identified, as demonstrated previously by several studies within the field of cardiovascular medicine^24,27,44–46^. Indeed, we were also able to delineate three phenogroups in our cohort of CRT upgrade patients, and only in one of them was CRT-D upgrade associated with a lower risk of all-cause mortality than CRT-P upgrade. We also recognized the importance of providing a solution for classifying new patients into the identified phenogroups, as that would allow others to validate and directly make use of our findings. To this end, we labeled the patients within the topological network based on their location, and then, using this newly labeled data, we trained an ML classifier, which we also made publicly available. Although our findings must be confirmed in future studies and the proposed ML classifier requires a thorough external validation, we may conclude that TDA, in conjunction with ML, holds the promise to optimize device selection and improve outcomes in patients undergoing CRT upgrades.

### Limitations

Besides its strength, our study has several limitations that should be discussed. First, the dataset we analyzed using conventional statistics and TDA was derived from a single center and included a relatively small number of patients. Thus, additional investigations should be conducted in the future to confirm our findings in larger, preferably multi-center cohorts of patients undergoing a CRT upgrade. Second, the retrospective nature of data collection bears several inherent limitations, such as the relatively high proportion of missing values, which forced us to omit several well-established prognostic markers (e.g., NT-proBNP) from our analysis. Third, patients were upgraded to a CRT-D or a CRT-P device based on the physicians’ clinical judgment and not in a randomized fashion, which may have resulted in selection bias (e.g., men with less deprived renal function were more likely to receive a CRT-D). Nevertheless, we also performed multivariable Cox regression analysis to partially countervail this bias. Fourth, post-mortem device interrogations were not performed, and cause-specific mortality data were unavailable; therefore, we could not investigate the differences in the rate of SCD between the groups. Last, although we trained an ML model to enable the classification of new patients into the TDA-derived phenogroups, we could validate it externally only in a small cohort of patients. Thus, further external validation will be required. To facilitate that, we made the source code as well as the best-performing model publicly available.

## Conclusions

In our cohort of patients with preexisting PMs and no history of VAs, upgrading to a CRT-D was found to be associated with a lower risk of all-cause mortality than upgrading to a CRT-P. By simultaneously evaluating multiple clinical features, TDA identified a phenogroup of CRT upgrade patients who were more likely to show additional benefit in terms of all-cause mortality from implanting a CRT-D instead of a CRT-P. We also trained and published an ML model that enables the risk stratification of new patients by assorting them into the TDA-derived phenogroups.

### Supplementary Information

Supplementary Information.

## Data Availability

The datasets analyzed during the current study are available from the corresponding author upon reasonable request.
