# Supplementary material for: Phenogrouping and risk stratification of patients undergoing cardiac resynchronization therapy upgrade using topological data analysis
Source: Sci Rep. 2023 Nov 23;13:20594. doi: 10.1038/s41598-023-47092-x (PMC10667223; doi:10.1038/s41598-023-47092-x)
Supplement: Supplementary file 1 — Supplementary Information. [file 41598_2023_47092_MOESM1_ESM.docx]

**SUPPLEMENTARY INFORMATION**

**Phenogrouping and risk stratification of patients undergoing cardiac resynchronization therapy upgrade using topological data analysis**

**Supplementary Table 1** Clinical characteristics of the intermediate-risk phenogroups

|  | **Lower region**  **n=48** | **Upper region**  **n=61** | **p-value** |
| --- | --- | --- | --- |
| Age, years* | 76.7±6.1 | 69.0±10.2 | <0.001 |
| Male* | 35 (73) | 47 (77) | 0.785 |
| CRT-D* | 14 (29) | 17 (28) | 1.000 |
| NYHA III-IV* | 14 (29) | 39 (64) | <0.001 |
| **Medical history** | | | |
| Atrial fibrillation* | 28 (58) | 26 (43) | 0.151 |
| Diabetes mellitus* | 18 (38) | 25 (41) | 0.863 |
| Hypertension* | 38 (79) | 44 (72) | 0.534 |
| Ischemic etiology of HF* | 43 (90) | 8 (13) | <0.001 |
| Myocardial infarction* | 38 (79) | 1 (2) | <0.001 |
| PCI* | 31 (65) | 5 (8) | <0.001 |
| CABG* | 18 (38) | 3 (5) | <0.001 |
| Time to upgrade, years | 7.5 (3.0-11.1) | 5.6 (1.9-10.5) | 0.612 |
| **Laboratory parameters** | | | |
| NT-proBNP, pg/mL (28) | 1,172 (834-2,288) | 4,894 (3,670-7,408) | <0.001 |
| Creatinine, μmol/L (77)* | 110 (90-133) | 135 (89-166) | 0.109 |
| GFR, mL/min/1.73m^2^ (77)* | 58 (45-74) | 49 (33-74) | 0.178 |
| **Echocardiographic parameters** | | | |
| LVIDd, mm (99)* | 52 (48-56) | 66 (62-71) | <0.001 |
| LVIDs, mm (88)* | 40 (37-45) | 57 (54-61) | <0.001 |
| LVEF, % (101)* | 35 (33-40) | 25 (20-30) | <0.001 |
| **Medications** | | | |
| ACE-I/ARB | 43 (90) | 54 (89) | 1.000 |
| Beta-blocker | 44 (92) | 53 (87) | 0.544 |
| Loop diuretics | 34 (71) | 52 (85) | 0.111 |
| MRA | 33 (69) | 45 (74) | 0.717 |
| Amiodarone | 11 (23) | 9 (15) | 0.399 |

*Variables used as input features in topological data analysis.

The value (in parenthesis) after a feature’s name indicates the number of patients with available data. If no value is reported, the given feature is available for all patients. Continuous variables are expressed as mean ± standard deviation or median (interquartile range), whereas categorical variables are reported as frequencies (n) and percentages (%). Phenogroups were compared using the Kolmogorov-Smirnov test for continuous variables and Chi-squared or Fisher’s exact test for categorical variables, as appropriate.

ACE-I – angiotensin-converting enzyme inhibitor, ARB – angiotensin receptor blocker, CABG – coronary artery bypass graft surgery, CRT-D – cardiac resynchronization therapy-defibrillator, GFR – glomerular filtration rate, HF – heart failure, LVEF – left ventricular ejection fraction, LVIDd – left ventricular internal diameter at end-diastole, LVIDs – left ventricular internal diameter at end-systole, MRA – mineralocorticoid receptor antagonist, NT-proBNP – N-terminal pro-brain natriuretic peptide, NYHA – New York Heart Association, PCI – percutaneous coronary intervention

**Supplementary Table 2** Performance of multi-class classifiers during internal validation

|  | **Accuracy** | **Balanced accuracy** | **Micro-avg.**  **precision** | **Macro-avg.**  **precision** | **Micro-avg.**  **recall** | **Macro-avg.**  **recall** | **Micro-avg.**  **F1** | **Macro-avg.**  **F1** | **Micro-avg.**  **AUC** | **Macro-avg.**  **AUC** |
| --- | --- | --- | --- | --- | --- | --- | --- | --- | --- | --- |
| **GBC** | 0.863  (0.813-0.913) | 0.867  (0.819-0.915) | 0.863  (0.813-0.913) | 0.866  (0.815-0.918) | 0.863  (0.813-0.913) | 0.867  (0.819-0.915) | 0.863  (0.813-0.913) | 0.865  (0.816-0.914) | 0.959  (0.945-0.973) | 0.948  (0.935-0.962) |
| $\boldsymbol{k}$**-NN** | 0.884  (0.851-0.917) | 0.889  (0.855-0.923) | 0.884  (0.851-0.917) | 0.888  (0.855-0.920) | 0.884  (0.851-0.917) | 0.889  (0.855-0.923) | 0.884  (0.851-0.917) | 0.885  (0.851-0.918) | 0.969  (0.954-0.984) | 0.960  (0.938-0.982) |
| **LogReg (L1)** | 0.825  (0.781-0.868) | 0.832  (0.790-0.874) | 0.825  (0.781-0.868) | 0.825  (0.779-0.871) | 0.825  (0.781-0.868) | 0.832  (0.790-0.874) | 0.825  (0.781-0.868) | 0.824  (0.779-0.869) | 0.948  (0.919-0.976) | 0.945  (0.926-0.963) |
| **LogReg (L2)** | 0.825  (0.759-0.890) | 0.834  (0.771-0.896) | 0.825  (0.759-0.890) | 0.839  (0.784-0.895) | 0.825  (0.759-0.890) | 0.834  (0.771-0.896) | 0.825  (0.759-0.89) | 0.822  (0.748-0.895) | 0.937  (0.903-0.972) | 0.926  (0.890-0.963) |
| **MLP** | **0.895**  **(0.851-0.938)** | **0.898**  **(0.854-0.942)** | **0.895**  **(0.851-0.938)** | **0.898**  **(0.852-0.943)** | **0.895**  **(0.851-0.938)** | **0.898**  **(0.854-0.942)** | **0.895**  **(0.851-0.938)** | **0.896**  **(0.853-0.940)** | **0.983**  **(0.980-0.986)** | **0.980**  **(0.976-0.984)** |
| **RF** | 0.849  (0.801-0.897) | 0.854  (0.805-0.903) | 0.849  (0.801-0.897) | 0.852  (0.809-0.896) | 0.849  (0.801-0.897) | 0.854  (0.805-0.903) | 0.849  (0.801-0.897) | 0.850  (0.804-0.895) | 0.964  (0.949-0.979) | 0.900  (0.944-0.976) |
| **SVC** | 0.863  (0.813-0.913) | 0.870  (0.822-0.919) | 0.863  (0.813-0.913) | 0.867  (0.819-0.915) | 0.860  (0.813-0.913) | 0.870  (0.822-0.919) | 0.863  (0.813-0.913) | 0.863  (0.812-0.913) | 0.968  (0.952-0.984) | 0.962  (0.939-0.986) |

Internal validation was performed with 5-fold cross-validation. Each performance metric is reported with a 95% confidence interval.

AUC – area under the receiver operating characteristic curve, GBC – gradient boosting classifier, $k$-NN – $k$-nearest neighbors classifier, LogReg (L1) – logistic regression with L1 regularization, LogReg (L2) – logistic regression with L2 regularization, MLP – multi-layer perceptron, RF – random forest, SVC – support vector classifier

**Supplementary Table 3** Clinical characteristics of the external validation cohort

|  | **Semmelweis cohort**  **n=326** | **External validation cohort**  **n=29** | **p-value** |
| --- | --- | --- | --- |
| Age, years | 73.8 (68.7-78.9) | 69.3 (62.7-74.4) | 0.005 |
| Male | 81 (25) | 19 (66) | <0.001 |
| CRT-D | 117 (36) | 6 (21) | 0.149 |
| NYHA III-IV | 157 (48) | 15 (52) | 0.862 |
| **Medical history** | | | |
| Atrial fibrillation | 176 (54) | 14 (48) | 0.692 |
| Diabetes mellitus | 122 (37) | 8 (28) | 0.394 |
| Hypertension | 250 (77) | 23 (79) | 0.927 |
| Ischemic etiology of HF | 163 (50) | 10 (35) | 0.159 |
| Myocardial infarction | 115 (35) | 5 (17) | 0.064 |
| PCI | 107 (33) | 5 (17) | 0.097 |
| CABG | 54 (17) | 5 (17) | 1.000 |
| **Laboratory parameters** | | | |
| Creatinine, μmol/L | 108 (87-142) | 88 (78-117) | 0.068 |
| GFR, mL/min/1.73m^2^ | 58 (44-76) | 60 (41-76) | 0.904 |
| **Echocardiographic parameters** | | | |
| LVIDd, mm | 30 (25-35) | 29 (25-30) | 0.225 |
| LVIDs, mm | 60.5±8.2 | 66.8±6.4 | <0.001 |
| LVEF, % | 49.9±9.7 | 56.1±7.1 | 0.006 |

Continuous variables are expressed as mean ± standard deviation or median (interquartile range), whereas categorical variables are reported as frequencies (n) and percentages (%). The characteristics of the Semmelweis and the external validation cohort were compared using unpaired Student’s t-test or Mann-Whitney U test for continuous variables and Chi-squared or Fisher’s exact test for categorical variables, as appropriate.

Abbreviations as in Supplementary Table 1.

**
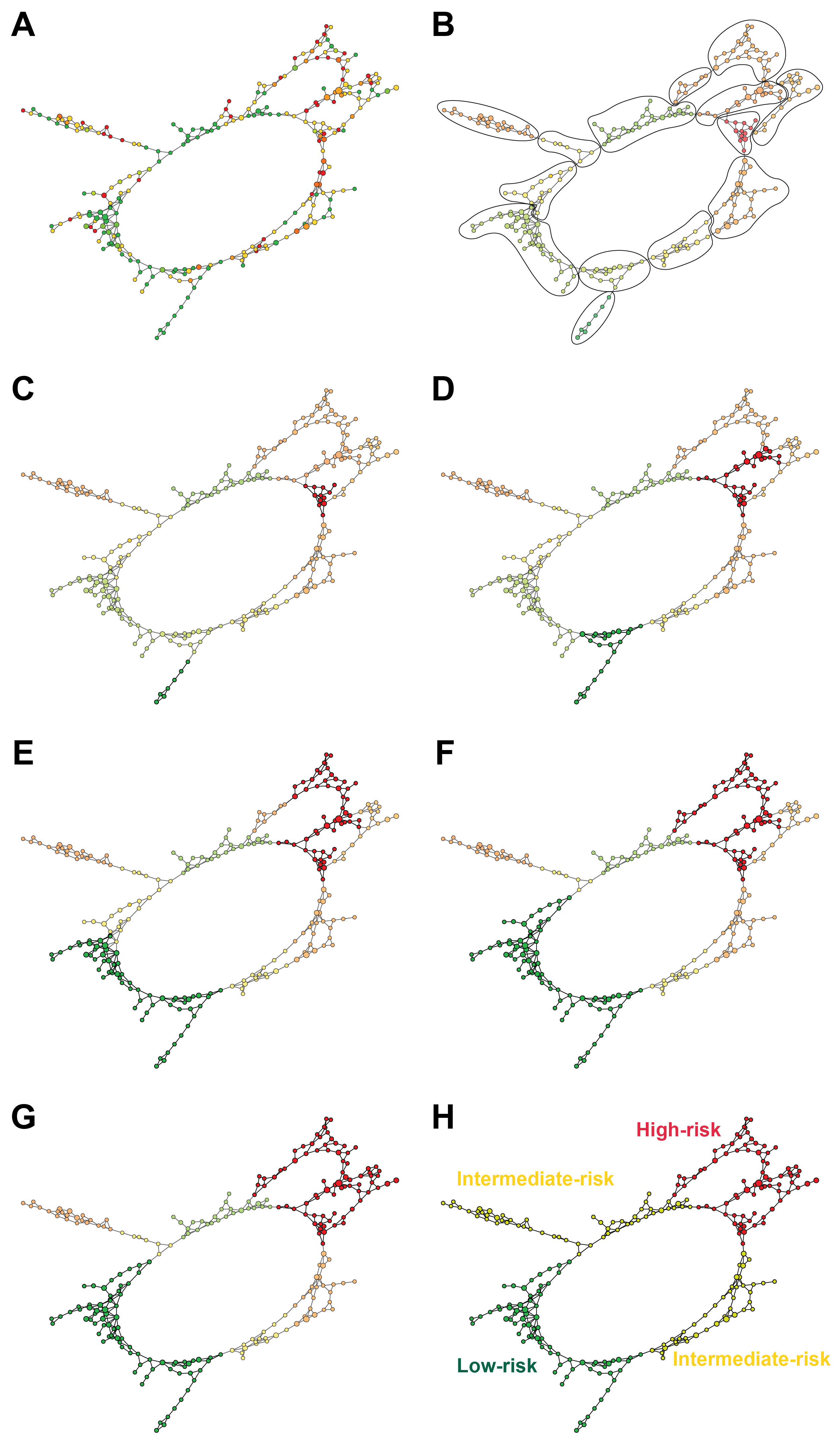
**

**Supplementary Figure 1** The steps of dividing the topological network into phenogroups

After generating a topological network and color-coding it based on all-cause mortality (A), we performed community autogrouping (B). This algorithm uses the Louvain Modularity optimization to find the best possible grouping of nodes with high intra- but low inter-group connectivity. In panel B, each autogroup is color-coded based on the mortality rate of the given group. Then, autogroups were sorted based on the survival rate of their members to identify the groups with the lowest (dark green) and highest (dark red) mortality rates (C). Next, each group was merged with an adjacent group having the most similar mortality rate (D). This step was repeated multiple times (E, F, G) until three phenogroups (i.e., low-, intermediate-, and high-risk phenogroups) with a nearly equal number of patients were created (H).

**
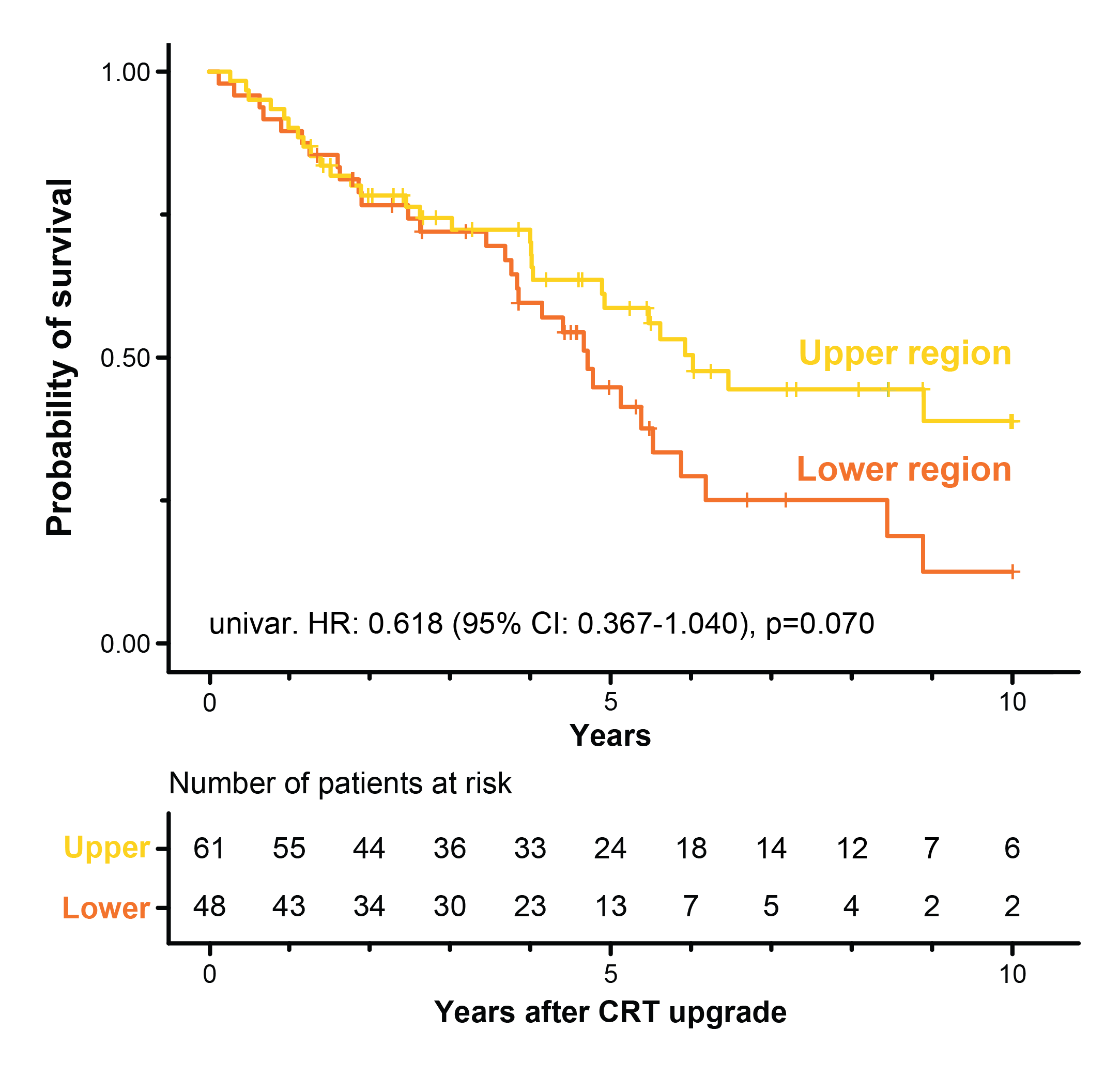
**

**Supplementary Figure 2** Kaplan-Meier curves depicting the survival of the upper and lower regions of the intermediate-risk phenogroup

Hazard ratios and 95% confidence intervals were calculated with univariable Cox regression.

CI – confidence interval, HR – hazard ratio

**
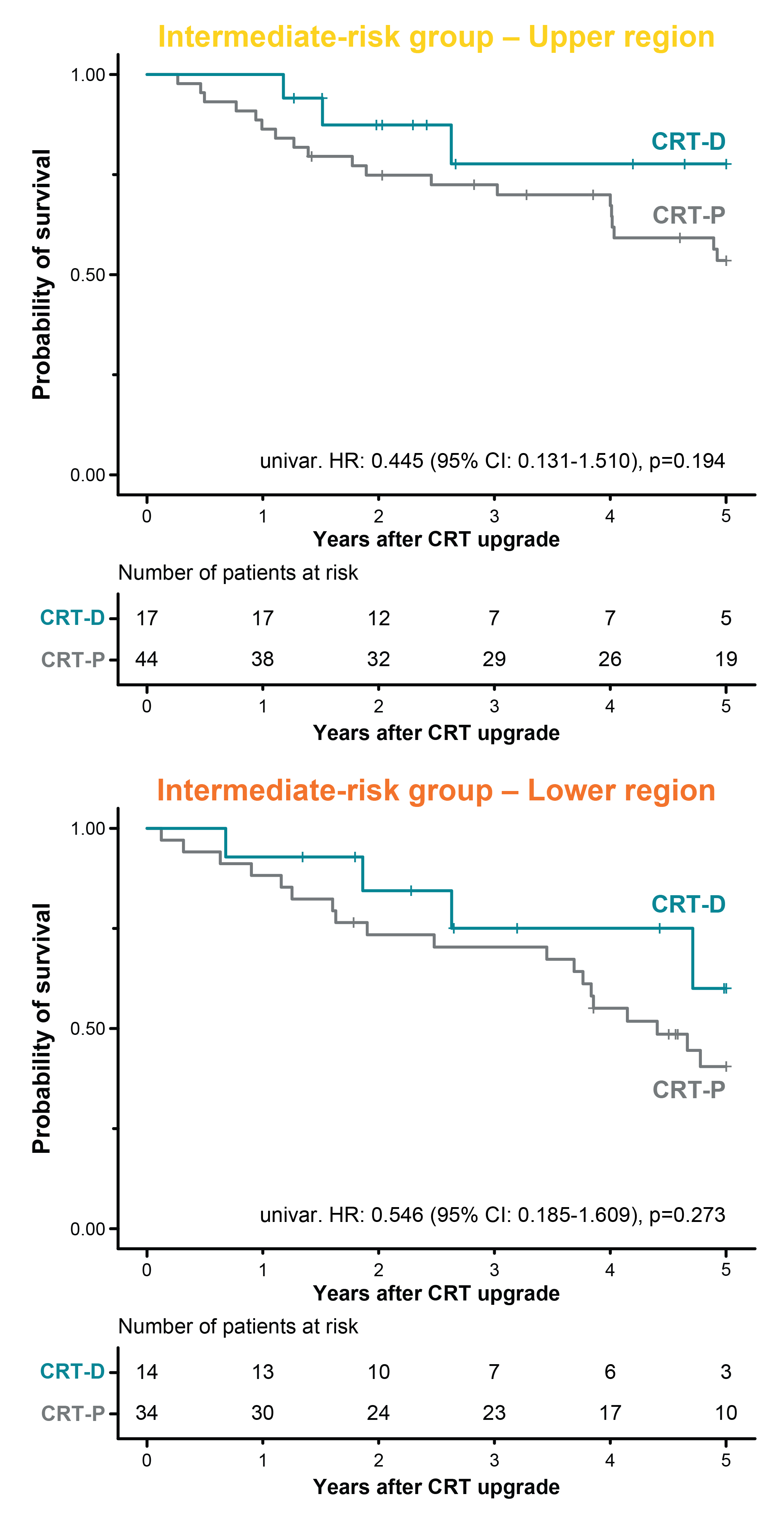
**

**Supplementary Figure 3** Kaplan-Meier curves visualizing the survival of patients who underwent an upgrade procedure to a CRT-D versus those who were upgraded to a CRT-P in the upper and lower regions of the intermediate-risk phenogroup

To assess the survival benefit of upgrading to a CRT-D compared with upgrading to a CRT-P, hazard ratios with 95% confidence intervals were calculated with univariable Cox regression.

CI – confidence interval, CRT-D – cardiac resynchronization therapy-defibrillator, CRT-P – cardiac resynchronization therapy-pacemaker, HR – hazard ratio
